# Mercury Exposure and Health Effects: What Do We Really Know?

**DOI:** 10.3390/ijms26052326

**Published:** 2025-03-05

**Authors:** Angelika Edyta Charkiewicz, Wioleta Justyna Omeljaniuk, Marzena Garley, Jacek Nikliński

**Affiliations:** 1 Department of Clinical Molecular Biology, Medical University of Bialystok, 15-269 Bialystok, Poland; 2 Department of Analysis and Bioanalysis of Medicines, Medical University of Bialystok, 15-222 Bialystok, Poland; 3 Department of Immunology, Medical University of Bialystok, 15-269 Bialystok, Poland

**Keywords:** mercury, toxicity, sources, environmental pollution, health effects

## Abstract

Mercury is widely used in medicine, agriculture, and industry. Meanwhile, according to the World Health Organization, it has been ranked as one of the ten most hazardous substances in the world, with the Agency for Toxic Substances and Disease Registry ranking it third. It has no known positive functionality in the human body, and even at low concentrations, it can have harmful long-term health effects, seriously affecting the healthcare system as well as posing a serious public health threat. This review focuses on the health effects of mercury and its major sources in the environment. We highlight its major toxic role in almost every possible aspect. Mercury and its forms, even in the smallest doses, cause numerous disorders to the body, including to the nervous system, the respiratory system, and the cardiovascular system. It can cause disorders such as various cancers; endothelial dysfunction; gastric and vascular disorders; liver, kidney, and brain damage; hormonal imbalances, miscarriages, and reproductive disorders; skin lesions; vision damage; and even death. The fact of such widespread use as well as its toxicity to the human body prompts further and in-depth research in populations of both low and moderate exposure. The constant controlling and monitoring of mercury use is a serious public health problem, requiring urgent attention and attentiveness from the governments of all countries and, in the long run, a rapid and concerted response. Thus, it is important to analyze in depth the impact of this highly toxic metal on the human body and to prepare the most precisely targeted public health interventions among all decision- and policy-makers.

## 1. Introduction

Mercury (Hg, Latin: Hydrargyrum) has been used and applied by the Greeks, Egyptians, Romans, and Indians for centuries, practically since ancient times (even 20,000–30,000 BC). Today, it is massively used, for example, in medicine, agriculture, and industry [1,2,3]. Under normal conditions, it occurs in a liquid state with a silvery-white hue and strong luster, resembling lead in appearance [2]. There are mainly three forms: elemental mercury (Hg^0^), inorganic mercury (IHg: Hg^+^ and Hg^2+^), and organic mercury (methyl—MeHg; ethyl—EtHg; and phenyl—PhHg) [3,4,5,6], with the MeHg form being the most dangerous and harmful to human health or even life [7].

Mercury is a naturally occurring element in air, water, and soil (the earth’s crust in the form of mercury sulfide (HgS—cinnabar, 0.05 ppm). It is considered by the World Health Organization (WHO) and the U.S. Agency for Toxic Substances and Disease Registry (ATSDR) to be one of the top three chemicals of public health concern, due to both its widespread use and toxicity to the human body, representing a sometimes problematic long-term disposal and health risk [8,9,10,11,12]. With an estimated 19 million people worldwide (4.5 million women, 600,000 children) at risk from its exposure [13], methylmercury is the most toxic organic mercury compound [3]. 

Also, Ogundipe and Obeng-Gyasi show a significant effect of major heavy metals on static load (AL), taking into account alcohol consumption and smoking in the U.S. National Health and Nutrition Examination Survey (NHANES) population [14,15]. Some studies evaluating the impact of heavy metals or toxic substances in the workplace have repeatedly indicated their impact on many diseases, including cardiovascular [16,17,18].

The purpose of this review is to analyze the most recent available literature on mercury and concentrate on the health effects of mercury and its main sources in the environment. We highlight its major toxic role in almost every possible aspect.

## 2. Strategy and Method of Selecting Articles

The following manuscript offers a unique aspect, presenting the multidirectional impact of mercury on public health, general health or lack thereof, environmental impact and, importantly, institutional and individual action or lack thereof. This multidirectional combination provides a holistic view of the mercury toxicity problem.

**Figure 1:**
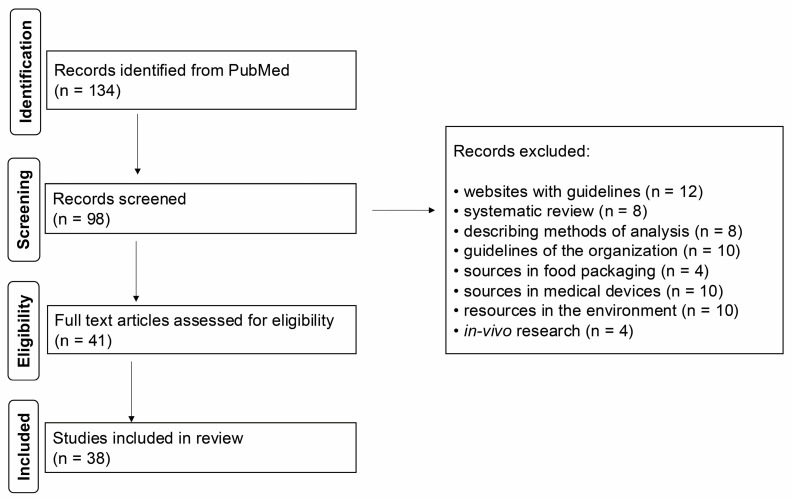
PRISMA diagram for literature search and screening.

The literature search followed the Preferred Reporting Item for Systematic Reviews and Meta-Analyses (PRISMA) guidelines [19], followed by systematic classification of selected articles (Figure 1). Accordingly, when searching the open-access PubMed database, we decided to select only the most recent literature from the last 10 years (from 1 August 2015 to 31 December 2024). The search was limited to English-language articles and only in open access using the following keywords: “sources of mercury food packaging”, “mercury medical products”, “mercury sources the environment”, “mercury health effects”, “mercury global guidelines”, “mercury test methods”, “mercury vs. *in-vivo* studies”, “mercury toxicity”, “mercury exposure”, “mercury poisoning”, “mercury”.

The search for articles was divided into six thematic categories: “sources in food/packaging”, “mercury in medical products”, “environment”, “health effects”, “analytical methods used to determine mercury”, “guidelines of various international organizations”. The “Health Effects” section was divided into smaller sections on the most important Hg-related risks and was the most important for the authors, where they were ultimately used to analyze 38 articles (Table 1).

## 3. Sources in Food/Packaging

Bioaccumulation of mercury (methylmercury) occurs when an organism contains higher concentrations of this element than the environment, such as found in fish and crustaceans [8,9,11].

On the packaging of cosmetic products, they are most often listed under the name: mercury, ethylmercury, mercuric oxide, mercuric iodide, mercuric chloride, phenylmercury salts, ammoniacal mercury, mercuric chloride amide, or as “poison” [49]. The all-popular fashion for skin lightening is influencing the demand for these products, forecast to grow from $11.8 billion by 2026 in Asia-Pacific and among dark-skinned populations in Europe and North America [8,49,50]. It is most commonly found in creams, soaps (e.g., antiseptics), mascaras, and eye makeup cleansers. Some of these products often pose serious health problems, often requiring hospitalization [1,20,49].

It is worth noting that properly preparing (e.g., cooking, frying) foods (here thinking mainly of fish) can significantly alter bioavailability and reduce its potential harm after consumption [7,51]. And, for example, consuming black coffee and green or black tea in addition to a meal reduces its bioavailability by up to 50–60% [52]. It is also undeniable that you can reduce the cardiotoxic effects of mercury by adding nutrients to your diet, such as long-chain unsaturated fatty acids and selenium [7,16,17].

Some stages of the food chain (i.e., processing and/or packaging) may contribute, to a greater or lesser extent, to the content of toxic elements in food, including mercury [11].

The World Health Organization has set a guideline for the content in drinking water in mg/L for Hg 0.006 [12]; meanwhile, the Codex Alimentarius (Food Code) in Commission Regulation (EU) 2023/915 of 25 April 2023 established a maximum level of mercury in food, depending on the product: in dietary supplements and salt, up to 0.10 mg/kg; selected fish, up to 0.30–1.0 mg/kg; in other fish, crustaceans, mollusks, and fish meat, up to 0.50 mg/kg [53]. Thus, in 2007–2008, in workers in the chemical, mining, and metal industries in southwestern Spain, a mercury-rich diet was shown to pose a serious risk and source of exposure for this industry sector [21].

Mercury is most commonly found in the following products:

- sharks, swordfish, mackerel, shrimp, canned tuna, salmon, catfish [9,22],
- predatory fish, shellfish [23,54],
- seafood containing mainly methylmercury [2,11,15,51],
- herbs and spices consumed in Europe (peppers, black bell pepper, basil, thyme, parsley) [55],
- cereals, rice, vegetables, fruits, oils and fats [4,11,23],
- mushrooms [2],
- dietary supplements [2],
- certain medications (teething powders, pain medications) [4,20],
- wild animals, meat, poultry [20,23].

## 4. Mercury in Medical Products

Mercury was traditionally and widely used in medical devices, such as thermometers and blood pressure devices. For nearly 200 years, it was used as dental amalgam (a filling material for treating tooth decay) [3,18,20,55]. And in the 16th century, its compounds were introduced into medicine and pharmacy by Paracelsus. Originally, it was an ingredient in medicines for the treatment of syphilis [2,3,24]. For a longer period of time, it was used at least as a diuretic, antiseptic, various skin ointments, or laxatives, and, in extreme cases, as a poison [3].

In the form of thimerosal (ethylmercury), it is used in small amounts as a preservative in some pharmaceutical products and some vaccines. This substance, however, is closely monitored and controlled by the WHO, the European Centre for Communicable Disease Control (ECDC), and the European Medicines Agency (EMEA), especially for vaccines. The two international organizations have consistently stressed that the amount of thimerosal does not pose a health risk [2,8]. All organizations confirm that there is no connection between vaccinating children with thimerosal-containing products and the incidence of autism or other brain diseases in children. Vaccine manufacturers in the U.S. have been phasing out its use as a preservative since 2000. Meanwhile, in Poland in 2009, the Sanitary and Epidemiological Council of the Chief Sanitary Inspector in Warsaw also issued an opinion confirming the lack of a connection between the use of vaccines with thimerosal and autism [2]. Currently, only some drugs allow for its removal from the body by chelation [22].

In an effort to gradually eliminate the use of mercury in medical devices, several countries (Albania, Burkina Faso, India, Montenegro, and Uganda) have joined forces to begin using alternative products, thus protecting healthcare workers and patients from harmful fumes. Digital replacements with equally accurate levels can be as much as 1/3 the price, which should also be an incentive to use them [50].

A Food and Drug Administration (FDA) report in 2004 provided insufficient data to support a connection between mercury release from dental amalgam and various patient complaints. In 2009, amalgam was additionally shown to be a valuable, cost-effective, and safe choice for dental patients. It was not until the 2013 Minamata Convention that the decision to restrict all forms of mercury use was confirmed [8,54,56]. Thus, in the U.S. alone, the rate of amalgam restorations has been shown to decrease from 6.29/100 patients in 2017 to 4.78/100 patients in 2019 [57]. But despite all of this, Tobias et al. [56], in their study, did not confirm the superiority of amalgam over composite restoration and therefore also support Minamata’s recommendation to use only composite resin materials. At the same time, Bjorklund et al. [25], in their study, confirmed that the removal of dental amalgam improved the health of patients with chronic fatigue syndrome (CFS). According to Webster et al.’s [58] analysis of the literature, no serious health consequences have been demonstrated among dental workers. Rather, the authors presented a problem resulting from the emission of amalgam fillings into the environment due to cremation of cadavers. Apparently, increased mercury concentrations have been observed in empirical studies, both in the crematorium workers themselves and in local residents.

It is notable that the European Union and other countries (e.g., Canada, the Philippines, the United States, a number of African countries—including Ghana, Nigeria, and Uganda) have laws banning mercury-containing cosmetics. Nonetheless, there are still a large number of countries (Bangladesh, China, Dominican Republic, Hong Kong, Jamaica, Lebanon, Malaysia, Mexico, Pakistan, Philippines, Republic of Korea, Thailand, and the United States of America) producing mercury-containing cosmetic products, bringing in tremendous financial profits but also taking a huge toll [49]. These dangerous cosmetic products, used by both men and women, are used to fade freckles, blemishes, age spots, and treat acne. Considering this, the 2013 Minamata Convention clearly set a limit for mercury—1 mg/1 kg (1 ppm) in such products [8,49,54,59]. In a study by Podgórska et al. [1] on 268 samples of personal care products/natural and conventional cosmetics, available in stationary and online sales, as well as in pharmacies and beauty stores, they found from 0.348 to 37.768 g/kg of mercury, with facial products containing significantly more than body products. The authors hypothesize that in the absence of a theoretically safe level for mercury, any concentration above zero is dangerous [1].

## 5. Environment

Naturally, mercury is released into the environment from volcanic activity, the state of rocks. It is also released from human activity, particularly from coal-fired power plants, residential coal combustion for heating and cooking, many industrial processes, waste incinerators, and the mining of mercury, gold, and other heavy metals [8,20,60,61,62,63,64]. After one use, it can be recycled for other necessary uses without further extraction, so reuse (especially not cyanide) should be promoted. The greatest impact on the population takes place in gold mining, where safe work practices are often forgotten [8,20,60,61]. Thus, workers in the metallurgical sector as well as mercury or gold mines are highly exposed, confirming the occupational factor as one of the main causes of mortality and exposure [6,7,58,65].

In addition to water bodies and in the air, its natural source is also found in soil (up to about 100 ppb), being a natural source for plants that assimilate it [12,20,26]. In the ecosystem, MeHg is easily transported through water, penetrating aquatic ecosystems, while it is poorly soluble there, which is why it is so commonly accumulated in fish [3,7,20].

Nevertheless, soils in Tibet, including the Jinmucuo Lake estuary, seriously exceed the concentration of this element (0.20 mg/kg), the most important ecological risk factor in the region. Its main sources have been geothermal activity, in-kind inputs, and atmospheric transmission [66]. Also, mercury is one of the most dangerous environmental pollutants with bioaccumulative actions in the environment [20,27,64]. The United Nations Environment Program (UNEP) in 2015 estimated global mercury emissions from human activities as high as 2200 tons in the atmosphere [67]. According to a 2019 UNEP report, the largest anthropogenic Hg emissions to air have increased, accounting for 70% of all global emissions since 2010, mainly covering three regions: East Asia, South America, and Sub-Saharan Africa [28].

Occurring naturally in the environment, it can be converted by bacteria into methylmercury [8,9,20]. Therefore, it is worth mentioning that the resistance of certain bacteria to Hg is quite a significant and important tool for reducing its level of contamination (bioremediation), providing a cheap and safe tool for restoring the contaminated environment [64].

Mercury is contained in many products, including the following:

- rechargeable batteries, batteries [3,20];
- measuring devices such as thermometers and barometers [3,20];
- electrical switches, switches and relays in appliances [20,68];
- lamps and lighting (e.g., certain types of incandescent light bulbs—fluorescent tubes) [3,20];
- non-electronic measuring devices [3,20,50];
- dental amalgam (for dental fillings—now phased out in many countries) [3,7,20,50,56];
- dental waste [7,50,64];
- incineration of medical, municipal waste [20,50];
- cosmetic products for skin lightening and other cosmetics, diapers, latex gloves [3,7];
- pharmaceuticals [3,50];
- paints (e.g., latex) [64];
- metallurgical components and all their activities (e.g., taps, pipes, drains) [64,68];
- fossil fuel emissions [7,20,54,64,68];
- cement and chemical production [64,68];
- chlor-alkali plants (e.g., chlor-alkali soda and caustic soda products) [54,64];
- explosives [7,20,54,64,68].

## 6. Health Effects

Mercury is toxic to human health, posing special risks at every level. It is most commonly found in various forms: elemental (or metallic), inorganic (e.g., mercuric chloride) and organic (e.g., methyl and ethyllortium). Each of the forms listed has different toxic effects [8,14,29,69]. It does not have any known positive functionality in the human body, and even at low concentrations, it can have harmful long-term health effects, causing headaches, limb pain, tooth loss, or general weakness at first, for example [11,12,21,30].

**Figure 2:**
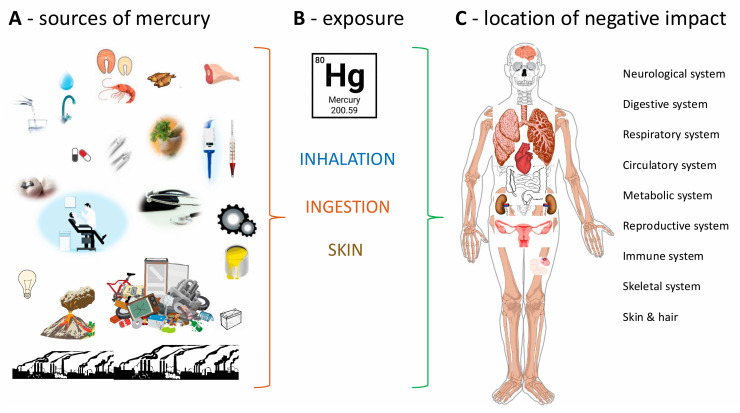
Sources of mercury (**A**), its exposure (**B**), and location of negative impact (**C**) on various parts of the human body. (**A**)—More information on sources is provided in Section 2, Section 3 and Section 4 (e.g., from food as well as from equipment or everyday household appliances). (**B**)—The forms of exposure (internal or external routes) are described in Section 5. (**C**)—The effect of exposure is described in the individual sections of Section 5.

Mercury causes numerous disorders, including nervous, respiratory, and cardiovascular disorders; endothelial dysfunction; various types of cancer; stomach and blood vessel disorders; liver, kidney, and brain damage; hormonal imbalances; miscarriages; skin lesions; vision damage; and even death (Table 2) [7,12,14,24,29,31,65]. Figure 2 shows the different sources of mercury (A), its type of exposure (B), and location of negative impact (C) on different parts of the human body. Also, the methylmercury form, already at a low enough dose, can cause neurodevelopmental, cardiovascular, and immunological problems [6,21,51,58,69]. Some of the illustrations in create Figure 2 were used from the free website Pixabay. The clinical manifestations of mercury poisoning depend on the form of mercury ingested, its dose, its duration of action, and the form of exposure (internal pathways or external, e.g., occupational, environmental) [2,3,6,26].

Methylmercury is mainly absorbed through the gastrointestinal tract and then distributed throughout the body. It also ends up in the brain, spinal cord, and central nervous system, where it can cross the blood–brain barrier. Being metabolized to inorganic mercury, it is excreted slowly through urine and feces. Its half-life depends on its form, while methylmercury itself is estimated to last from 40 to 80 days in the human body [2,14,20]; although some authors suggest up to 28 years in the brain alone, this depends on diet, genetics, and ethnicity [6,20]. It is likely that the half-life of MeHg in the sexes differs, representing 8.4–81.6 days in men and 8.6–78.9 days in women. Thus, the elimination process of this organic mercury from the body is quite long. Meanwhile, the half-life of Hg itself from the vapor to the blood accounts for 2 to 4 days, although most will be excreted, 90%, in urine or blood [6]. Nails and hair are good biomarkers of exposure to organic Hg, indicating its longer exposure [7,21]. Most often in hair, the Hg dose is up to 300 times higher than in blood as a result of sulfur, which easily combines with MeHg [6]. In contrast, in blood or urine, mercury persists for a much shorter time, confirming rather fresh contact [7].

Meanwhile, among hormones, insulin, estrogen, testosterone, and epinephrine are the most affected. The pituitary gland, together with the thyroid gland, have a significant affinity for mercury accumulation. As a result, hormonal areas are inhibited or malfunction, leading to at least hypothyroidism, impaired body temperature control, thyroiditis, and depression [25,26,58,65].

The most common exposure is through inhalation, ingestion, and skin [10,21] and occupational exposure [32]. One of the most commonly mentioned diseases in the literature is Minamata disease (*Minamata-byō*), otherwise known as mercurial (mercurialism), severely damaging the nervous system [2,29,32].

### 6.1. Digestive System

Mercury is absorbed as much as 7% by the gastrointestinal route and then transported to all body tissues, while the MeHg form is absorbed as much as 85–95% [2,3,6,20]. Immediately after ingestion, it is absorbed by epithelial cells, sometimes causing digestive disorders. Most often inhibiting the production of digestive trypsin, chymotrypsin, pepsin, xanthine oxidase function, or even dipeptidyl peptidase IV [20]. Ingestion of mercury compounds causes quite severe salivation, abdominal pain, vomiting, indigestion, stomach ulcers, bloody diarrhea, characteristic blue-purple borders appear on the gums, necrosis of the intestinal mucosa, and, in extreme cases, even death. There may also be a burning sensation in the esophagus [2,6,20].

Also, Tian et al. [4], wishing to better understand the toxicity of this element, describe in their review the effects of IHg and MeHg on the gut and their contribution to extraintestinal effects (including intestinal barriers or gut microbiota). Unfortunately, ingestion of contaminated food destroys the intestinal flora [20], and most often, prolonged exposure disrupts liver and appendix function [33]. It can also cause nausea, lethargy, or even jaundice lasting up to six months. Unfortunately, there are known cases where patients knowingly swallowed large amounts of mercury for suicidal purposes, so much so that the rapid response of healthcare professionals (mainly through lavage, endoscopy, colonoscopy) restored to function, albeit often with serious consequences in the subsequent state of health [5,33].

### 6.2. Respiratory System

Mercury metal vapors, as they are quite volatile, are absorbed by the respiratory system by almost 80% [2,70]. Exposure to mercury can lead to an inflammatory reaction in the lungs or airways, reducing lung function and making breathing difficult [13,26]. Meanwhile, acute poisoning by its vapors can result in pneumonia and bronchitis, pulmonary fibrosis, chest pain, and angina, leading to death, due to respiratory failure [2,20,34,69]. Toxic fumes from the vaporization of mercury or the burning of materials with its composition in workers can cause various pulmonary conditions (such as Young’s syndrome) in workers [20,65].

A study of more than 4300 children aged 7 to 8 years from Korea found that increased mercury in the blood, even at low exposure levels, was associated with asthma [34]. Meanwhile, another study in adults from China’s Shandong Province, confirmed the connection between blood mercury concentrations and changes in lung function. It also connected this study to the frequency of fish consumption, indicating that the incidence of asthma was increased in those consuming fish 1–2 times a week [13]. Remarkably, a study conducted, this time in Germany, in three regions on more than 1000 children aged 5 to 14 years, found no connection between mercury exposure and asthma. The authors nevertheless suggest additional testing, including even prenatal evaluation as well as early exposure [35].

Meanwhile, in a cohort of adult patients with chronic obstructive pulmonary disease (COPD), it was shown to be higher in the blood, which could be related to impaired lung function [30]. Another study, The Korea National Health and Nutrition Examination Survey (KNHANES), conducted between 2005 and 2016, found that higher mercury concentrations were associated with airflow obstruction among the adult population [72].

### 6.3. Reproductive System

Mercury also induces pathophysiological changes along the hypothalamus–pituitary–adrenal and gonadal axis, which, in turn, can affect reproductive function. There is then a change in the circulating levels of lutein-stylizing hormone (FSH), luteinizing hormone (LH), estrogen, progesterone, inhibin, and estrogens [20,36]. Some studies confirm reduced fertility in people exposed to mercury, such as among dental assistants [71,73]. In men, it most often adversely affects spermatogenesis, testicular weight, and erectile dysfunction. In women, meanwhile, ovarian dysfunction, painful and irregular menstruation (including short, long, or irregular cycles), tilted uterus, or premature menopause due to disturbed estrogen and progesterone levels [20]. Also, symptoms of mercury toxicity can cause sexual dysfunction [58], thus disrupting at least reproductive potential.

Mercury poses a particular threat to the development of the child in utero and in early life. Its main exposure to methylmercury for the fetus is the risk to the unborn child [8,9,49]. Unfortunately, it has also been associated with the occurrence of miscarriages, spontaneous abortions, low birth weight, and even stillbirths [20,74].

Regrettably, some fish contain methyl mercury, which can significantly harm the developing fetus. Thus, recommendations have been made by the government in the U.S. by Food and Drug Administration and Environmental Protection Agency in 2004, for women of childbearing age to reduce their consumption of fish or shellfish, with the goal of avoiding risks to the unborn child or the just-developing nervous system [6,9,18]. 

### 6.4. Development and Its Potential Disorders

It is known that even a small amount of mercury exerts neurotoxic or immunotoxic effects, especially during early development, which is why it is so important to understand its effects on the short-term and long-term health of the offspring. Studies of its levels in cord blood repeatedly confirm its effects on DNA methylation (DNAm). Prenatal exposure to Hg has been connected to significant abnormalities in the child’s neurodevelopmental and cognitive abilities (e.g., delayed learning and memory deficits), with serious lifelong consequences. Unfortunately, the placenta will not protect against the toxic effects of this element, which easily crosses its barrier and enters the developing fetus with great facility [2,4,18,22,51]. Since the placenta is ideal for faster transport of MeHg, it has been shown that its concentration in fetal blood reaches about two times that of the mother alone. Thus, in this case, the ideal biomarker seems to be umbilical cord blood and tissue, as well as the placenta itself [6]. The most sensitive to its toxic effects is the metabolically immature fetal brain, resulting in cerebral and psychomotor paralysis in the latter stages of its development [20,69,75]. Disappointingly, the fertility autopsies performed also showed cerebellar hypoplasia, abnormal numbers of nerve cells in the cerebral cortex, also neuronal migration and layered organization, a rather pronounced decrease in total brain mass as a consequence of MeHg [20,32,38].

Cohen et al. [18] thoroughly presented in a manuscript prenatal MeHg exposure and cognitive effects in comparison to both their findings and those observed in the literature. They pointed out how, unfortunately, it can affect lowering a child’s intelligence quotient and other developmental behaviors that will be reflected in the general population in the long term. It is not uncommon to confirm that even low prenatal exposure to mercury can be positively associated with IQ [6].

Regrettably, in low- and middle-income countries, mercury exposure showed a higher incidence of spontaneous abortions, stillbirths, premature births, low birth weight and apparent congenital anomalies among the 961 Tanzanian women surveyed in 2015–2016 [27]. A study conducted in Mexico City confirmed the effects of prenatal mercury exposure on both DNA methylation and gene-specific, as well as mRNA expression [22]. Observation of 408 pregnant women aged 16 to 46 years in 2020, living in rural and urban areas in Suriname from northeast coast of South America, showed rather interesting results regarding both the baby and the birth action. The authors observed a significant incidence of preterm labor (15.1%), small baby size at gestational age (1.2%), low birth weight, and low Apgar score (3.8%) [37].

### 6.5. Immune System

Even the short-term exposure to mercury can cause serious health consequences, including immunotoxic effects, autoimmune dysfunctions, or various types of inflammation, inducing further pathological changes in the body [26,65]. Undigested mercury-laden residues enter the bloodstream, inducing various types of reactions from the immune system and consequently reducing its performance in infections. Also, people with lower immunity are more prone to allergies, asthma, autoimmune symptoms (e.g., rheumatoid, Crigler–Najjar syndrome (CNS), amyotrophic sclerosis, arthritis, multiple sclerosis) [20,26]. Constant exposure to mercury impairs the immune defense system, while the MeHg or IHg form damages cells and can induce apoptosis [54,58]. Individuals with low susceptibility even the shortest and smallest exposure to mercury can induce systemic/local inflammation. Meanwhile, in patients with autoimmunity, it can severely exacerbate the autoimmune response [26].

Among the quite useful and effective biomarkers in autoimmunity just induced by Hg have been found to be inflammatory pathways assessing cytokine regulation [26]. There are also other studies confirming correlations between mercury exposure and the production of pro-inflammatory cytokines, i.e., interleukin 1 (IL-1), tumor necrosis factor (TNF), and interferon-alfa (IFN-a) [13,26]. Many pro-inflammatory pathways and factors have so far been confirmed in animals and in vitro [26].

### 6.6. Neurological System

Mainly targeting the nervous system, mercury interferes with cellular processes (i.e., glutathione homeostasis, enzyme inhibition) [2,51,58]. Neurological and behavioral disorders are most often observed after inhalation, ingestion, or dermal exposure to various mercury compounds (the MeHg form is absorbed up to 10% in the brain), posing a serious public health problem. It is then manifested by tremors (including a specific change in handwriting to induced hand tremors), memory loss, insomnia, anxiety, depression, peripheral neuropathy, headaches, impaired concentration, hallucinations, personality changes, cognitive and motor disturbances, and neuromuscular response. Mild-level exposure (subclinical nervous system toxicity) can only be observed in exposed workers when the mercury level in the air is 20 g/m^3^, or when it is accumulated in higher amounts for longer, even over several years [2,8,20,26,49,51,69]. By accumulating in the brain, it damages nerve cells very seriously and thus increases the risk of developing multiple sclerosis, neurobehavioral defects, or anorexia [26]. However, it has unfortunately been shown to have negative effects on other centers, including special sensory systems (e.g., retinopathy, visual neuropathy, loss of hearing or vision, reduced sense of smell, abnormal touch) [20,26].

To date, mercury itself as well as its derivatives are of great concern, already started in fact by three incidents: two in Japan (in the 1950s and 1960s) in Minamata Bay and along the Agano River in Nigiata as a result of fish consumption or industrial waste deposition. Another incident occurred in Iraq in the early 1970s after ingestion of grains with fungal MeHg [3,18]. Unfortunately, in the long term, it negatively affected the CNS in exposed pre-born children [18].

Methylmercury can affect the function of microglia, astrocytes and oligodrocytes, and mechanisms with potential effects on the brain for some selected neurodegenerative diseases and neurodevelopmental disorders. As it readily accumulates in the brain, it can often lead to impaired health of neurons and their transmitters, neurodevelopmental disorders (e.g., autism spectrum disorder and neurodegenerative diseases: Alzheimer’s, Parkinson’s) [10,26,29,39]. Mercury, after crossing the brain, forms mercury salts, while on inhalation, it gradually transforms into mercury ions in the brain, remaining for a long period (up to 4 months) [49]. Astrocytes accumulating act as a reservoir for methylmercury in the brain, and its excessive accumulation causes swelling of astrocytes, causing neuronal damage. Meanwhile, MeHg induces pro-oxidant activity in neurons through two mechanisms: generation of ROS and antioxidant defense. It disrupts calcium homeostasis and neurotransmitter metabolism. It has been confirmed that TNF-alpha levels increase with Hg exposure, inducing neuroinflammation and cell death, causing symptoms like those of Parkinson’s disease [10,26]. Meanwhile, Minamata disease severely damages the nervous system, especially the brain, impairs vision, motor coordination, leads to intellectual disability, and, eventually, even death through excessive accumulation of methylmercury and dimethylmercury in the body [3,11,29,78].

A study from the NHANES series (2017–2018) outlines a number of factors that also affect depression, including through the impact of mercury and its effects. Researchers from the U.S. assessed the comprehensive impact on mental health, obtaining blood levels of 0.80 g/L in 153 women from 50 states. Its increased exposure is associated with fewer depressive symptoms, likely a result of the omega-3 fatty acids present in fish [15] or the antioxidant protective effects of selenium [40,51]. Researchers believe it may alleviate the effects of methamphetamine in depression, especially in women [15].

### 6.7. Circulatory System

Some studies confirm the effects of even low amounts of mercury and its derivatives on cardiovascular diseases (CVD), including, for example, hypertension, heart rate variability, vascular atherosclerosis, ischemic heart disease, myocardial infarction, myocardial paroxysmal activity, albeit cardiac autonomic function, or probable stroke. It is estimated that 5% of methylmercury is absorbed into the blood, disrupting the heart rate and function of the entire cardiovascular system [3,7,30,40,58]. Although, Chen et al. [40] did not confirm the association between mercury and the incidence of ischemic stroke in a population with low as well as moderate exposure in their The *Reasons for Geographic and Racial Differences in Stroke* (REGARDS) study in black and white Americans aged 45 years. However, the theory that the mechanism of Hg toxicity may affect endothelial dysfunction as well as thrombosis, consequently disrupting myocardial function, is also beginning to connect [7,26,65]. Meanwhile, Hg has been shown to increase the risk of developing Kawasaki disease [6,26].

Its accumulation in the heart contributes to cardiomyopathy, with levels in the heart tissue of those who died up to 22,000 times higher than those of those who died from other heart diseases. Mercury is also related to possible anemia (hemolytic anemia and aplastic anemia), as it competes with iron for binding to hemoglobin, leading to impaired hemoglobin formation [20].

There is a growing body of evidence showing the effects of heavy metals (as an environmental factor) on cardiovascular disease [7,41]. Researchers using data from NHANES between 1999 and 2018 from more than 5000 people with high residual cholesterol showed that mixed metals (including mercury) may be a potential cause of increased HRC risk [41]. Jung et al. [42] also evaluated the lifestyle of students from Gyeonggi-do in China, in conjunction with exposure to this toxic element without obtaining spectacular results or major correlations. Nonetheless, there is insufficient evidence/accepted biological explanation and a wide range of MeHg exposures in primary studies confirming the relationship, with dose as well as quantity affecting cardiovascular incidents [7,76,79].

It is noteworthy that omega-3 fatty acids, selenium (antagonistic effect), and vitamin D may be highly beneficial for protective cardiovascular effects, also reducing the toxic effects of mercury [7,40]. Meanwhile, the Prevention with Mediterranean Diet (PREDIMED) study in about 260 Spaniards did not confirm that regular fish consumption increases the risk of cardiovascular disease. On the contrary, Downer et al. [76] further maintain the recommendation of consuming at least 1–2 servings of fish per week for its protective effect on CVD.

### 6.8. Metabolic and Other Diseases

The kidneys are the most common route of excretion of toxic metals, including Hg [43,60,61]. They are uniquely susceptible to its toxicity; meanwhile, up to 50% of their function can be asymptomatic, making it problematic in the long run to detect their dysfunction as well as recover fully [2,43,69]. Mercury exposure can lead to kidney disease, increased levels of gamma-glutamyltransferase (a biomarker of liver function), indicating possible liver dysfunction [36]. It can also influence the development of non-alcoholic fatty liver disease, among others. Unfortunately, the presence of Hg can severely disrupt homeostasis, interfering with the absorption, distribution, and utilization of essential elements and, in the long run, leading to oxidative stress, inflammation, and the onset of various liver diseases [3,31]. A study from NHANES for 2017–2018 showed positive interactions between NAFLD and Hg [31]. 

The abnormal function of renal function reported most often includes several phases, ranging from increased protein in the urine to renal failure [8,59]. Constant exposure can cause at least renal syndrome, tubular disruption, secondary focal segmental glomerulosclerosis, renal group proteinuria, syncretic nephrotic syndrome, glomerular disease, and membranous nephritis. By reducing cortisol production, mercury induces a strong increase in adrocorticotropic hormones, consequently leading to adrenal hyperplasia. As a result of the adrenal atrophy that occurs, it can contribute to the development of Addison’s. It has also been shown to be susceptible to malfunctioning pancreatic function, causing interference with normal biological function and reduced blood glucose regulation [20]. An interesting aspect is the rate of mercury excretion by the kidneys, where women have been observed to have an accelerated metabolism adequate to that of men [13]. A similar trend of increased Hg concentrations in boys than in girls has been observed among adolescents in virtually all epidemiological analyses [23].

There is also emerging evidence of mercury exposure being linked to obesity or increased blood glucose levels [6]. And as hypothesized by Roy et al. [54] based on a review of epidemiological data from 25 original papers, its exposure may lead to the development of diabetes and multiple sclerosis, although there is insufficient evidence and a causal association.

Meanwhile, elevated serum Hb levels (3.11 g/L) were detected in Korean hybrids [43], just as people near abandoned mines showed elevated blood Hg levels (3.35 g/L) [80]: in the Canadian Health Measures Survey (CHMS)—0.71 g/L [81]; in German Environmental Survey 1998 (GerES III)—0.58 g/L [47]. Meanwhile, in the U.S., the NHANES (2017–2018) urinary Hb level was 0.18 g/L [82], while a 4-fold higher level was found in Koreans (3.11 g/L) [43].

### 6.9. Skeletal System

Further inconvenient information regarding this toxic element includes its effects also on bone defects, accelerating at least bone mineral density loss and osteoporosis. In addition, estrogen deficiency and disrupted metabolism reduces the development of the bone itself [24,44,45]. Mercury is also deposited in bone tissue and cartilage probably by incorporating its ions in place of calcium ions into carbonates or hydroxyatite. Nevertheless, there is still a lack of complete information on the osteotoxic effects of Hg on bone tissue. Insufficient full and complete data means that often even the results obtained are below the detection limit of the measuring device [24]. Pamphlett and Jew [44] concluded that exposure to this toxic element may play an important role in the pathogenesis of joint and connective tissue disorders, mixed connective tissue disorder, osteoarthritis, rheumatoid arthritis, systemic sclerosis, lupus erythematosus, fibromyalgia, and Sjogren’s syndrome, among others [65].

A study of 100 patients (2005–2008) diagnosed with systemic sclerosis in France showed a significant association between mercury exposure in women working with electronic devices, batteries, and fluorescent lights [83]. Meanwhile, authors from Mexico showed that Hg does not invade the bones but instead localizes to the bone matrix [76]. A U.S. study in more than 260 patients with systemic lupus erythematosus found that its incidence was associated with occupational exposure to mercury [84].

### 6.10. Skin

Organic and inorganic mercury compounds are absorbed through the gastrointestinal tract, skin, and sweat and sebaceous glands of the skin [5,26,70]. Hg poisoning manifests as redness, rash, facial swelling, or even excessive sweating, among other symptoms. The reaction on the skin can affect the hair, making it dry, porous, brittle, thinning, and matted, while inhibiting the growth phase [1,20]. With hair concentrations > 2 g/g, cardiovascular incidents of all kinds increase, while a dose above 4 g/g has been connected to CVD mortality [7].

Inorganic forms of mercury are corrosive to the skin in particular (e.g., causing skin rashes, redness, skin discoloration, scarring, reduced skin resistance to bacterial and fungal infections) and to the eyes. Unfortunately, a common and dangerous practice to date related to the risk of exposure to mercury salts is through skin bleaching or whitening to inhibit melanin production. However, products containing it have been banned in many countries, and in others, they are strictly controlled in terms of advertising, including their availability. Used cosmetics pose serious public health risks, including to children who may be exposed to breast milk, and food chains may be contaminated when cosmetics are washed down the drain [8,49,50,59].

Both metallic mercury and mercury salts (HgCl_2_) have the ability to penetrate through skin cells of the plasma membrane, as well as through the intercellular layer via the lipid bilayer and through the stratum corneum via the transfollicular layer. Its prolonged exposure causes accumulation in the skin and outgrowths, mainly in nails and hair [20,59].

In the meantime, studies from the National Health and Nutrition Examination Survey 2003–2006 and 2009–2014 in more than 16,000 participants aged 20 to 80 years showed no association between Hg and the incidence of psoriasis. Instead, the authors suggest a lack of overestimation and that it is more pronounced in people with severe psoriasis who also lead less healthy lifestyles, including alcohol consumption or smoking [48]. Similarly, the KNHANES study (2005–2016) in a Korean adult population of more than 18,000 found no association between blood mercury levels and any of the three allergic conditions analyzed, e.g., asthma, allergic inflammation, atopic dermatitis [72].

### 6.11. Cancers

It is worth mentioning that the International Agency for Research on Cancer (IARC) and the U.S. Environmental Protection Agency (USEPA) have classified mercury as a probable human carcinogen (Group 2B). And in 2022, mercury was leading among the most toxic and harmful to health, along with lead. Exposure to Hg is correlated with the incidence and location of various cancers, such as kidney, liver, stomach, thyroid, gallbladder, colon, prostate, breast, glioma, lung, basal cell, melanoma, squamous cell, and non-melanoma skin cancer [12,28,58,61,77]. It is also believed that it can be a factor in mononucleosis, participating in leukemia, such as with Hodgkin’s disease [20]. Although there are still sometimes conflicting data about the exposure itself as well as the risk of cancer, information about genotoxic effects is growing. It is even the low dose that can induce a proliferative response in both normal and cancer cells through interference with estrogen receptor, extracellular signal-regulated kinases (ERK1/2), c-Jun N-terminal kinases (JNK), NADPHoxidase, and potentially Transcription Factor (Nrf2) [28]. Kim et al. [77], in their study of 5213 residents near industrial complexes from South Korea, showed that mercury was associated with thyroid cancer and significantly affected red blood cell index status. American researchers, meanwhile, evaluating the potential exposure of both mercury itself and its various forms, observed an increased incidence of non-melanoma skin cancer (NMSC), using data from the NHNES (2003–2016) [46].

### 6.12. Molecular Aspect

Mercury affecting various molecular mechanisms, such as oxidative stress, mitochondrial dysfunction, activation of inflammatory pathways, alteration of gene regulation sequences by DNA in newborns, and disruption of cell signaling reflects its neurological actions at different levels [10,20,22,29]. It impairs the antioxidant system by increasing lipid peroxidation or inhibits antioxidants such as catalase and glutathione [58]. And the MeHg or IHg form damages cells such as pancreatic cells [54,65], as well as human embryonic kidney (HEK) and thyroid cell lines [58]. 

Hg induces apoptosis in neurons by affecting the membrane potential and the release of cytochrome C into the body, so that it contributes to neurodegenerative diseases. Mercury may be responsible additionally for inflammation or necroptosis [10,22] and increases the levels of rhodamine oxygen species having the ability to act as a catalyst for Fenton-type reactions [3,20,28]. 

Undoubtedly, genetic as well as epigenetic factors influence Hg exposure and associated health risks. There will also be information confirming that epigenetic mechanisms are very important in contact with this element. For mercury, as a highly toxic substance, is an established developmental toxicant that can, in the long term, affect the delay between exposure and a given chronic disease [6,26,44,68].

A very interesting aspect of in vivo studies was addressed in an analysis of the daily release of mercury vapor from amalgam restorations in patients from saliva and urine. No patient showed any effect on mercury levels dependent on the type of amalgam [85]. Meanwhile, chronic pot exposure has been studied in vivo on mice, confirming that it worsens atherosclerosis and mitochondrial function, stress, blood cell profile and disrupts morphological, metabolic, or immune status. This study could be very useful for future analysis on humans [86]. Another study also suggests that even low doses of mercury and its forms disrupt pancreatic function by affecting the development of diabetes in in vivo studies in lab animals [87]. It has also been shown that under in vivo conditions, the inorganic form of Hg can methylate to MeHg [88].

## 7. Analytical Methods Used to Determine Mercury

A crucial aspect of any mercury analysis is the use of an appropriate technique for its identification [89,90].

As noted by Chirita et al. [89], the appropriate method should be used appropriately for the sample to be collected and in accordance with the requirements of multiple directives in a given region of the world. The authors emphasize that standard and common laboratory methods should be developed as much as possible. 

Meanwhile, the cold vapor generation high-resolution continuum source quartz tube atomic absorption spectrometry (CVG-HR-CS-QTAAS) method for mercury determination is widely used in the analysis of human samples while using CVG with HS55-manual in food, environmental, and (bio)polymer matrices. Currently, very well-known and widespread methods are based on atomic absorption spectrometry (AAS), atomic fluorescence spectrometry (AFS), inductively coupled plasma optical emission spectrometry and mass spectrometry (ICP-OES, ICP-MS), laser-induced breakdown spectroscopy (LIBS), X-ray fluorescence spectrometry (XRF), and optical emission spectrometry. They are used in various microplasma sources and have been widely used for the determination of Hg [89]. The gas chromatography (GC) method is one of the most widely used techniques due to its high separation efficiency [91,92]. Authors from China have developed an improved headspace solid-phase microextraction with gas chromatography–triple quadrupole mass spectrometry (HS-SPME-GC-MS/MS) method for speciation of Hg^2+^, MeHg, and EtHg in water samples in terms of sensitivity, accuracy, and precision.

The application of gold nanoparticle (AuNP) in the selective detection of Hg^2+^ in water samples is quite interesting, where satisfactory and sensitive results were obtained [93]. 

Favilli et al. [90] evaluated a high-performance liquid chromatography (HPLC) method with their sensitive determination by Inductively Coupled Plasma Mass Spectrometry (ICP-MS). It determines multiple elements in a single analytical run and combines separation with detection systems and versatility in a very simple way. In addition, it has a highly sensitive detector. As the authors point out, this accuracy and sensitivity depends on the initial sample procedure. The HF-LPME-HPLC-ICP-MS ionic liquid-based coelution method is a fairly simple and sensitive technique. It is possible to carry out oz-determination of trace amounts of mercury in water samples and will also have high reproducibility and accuracy. The authors have developed a novel and fairly simple hollow-fiber-assisted liquid phase microextraction (HF-LPME) method for the pre-concentration and determination of monomethylmercury (MMHg) and inorganic mercury in environmental water samples. They combined this using high-performance liquid chromatography with inductively coupled plasma mass spectrometry (HPLC-ICP-MS) [94].

However, Inaudi et al. [95] have analyzed and refined laboratory procedures in a very precise way in fish samples. They additionally proposed the use of CYXAD cartridges, which can be used even before proceeding to long extraction for DMA analysis. 

Subsequent analyses in fish and water samples used a new effective strategy based on magnetic solid-phase extraction (MSPE) and dispersive micro solid-phase extraction (DμSPE) to preconcentrate Hg^2+^ [96]. Meanwhile, in a study of fish samples, Bozorgzadeh et al. [92] suggested the use of the (DµSPE) method in combination with an microwave digestion combined with an inductively coupled plasma optical emission spectroscopy (ICP/OES) procedure for Hg sorption onto GO/Fe_3_O_4_/pectin sorbent because of the well-ground accuracy and the precision. 

## 8. Guidelines of Various International Organizations

The World Health Organization has ranked mercury as one of the top ten most hazardous substances in the world, with the ATSDR ranking it third [6,22,28]. On January 19th, 2013, there was an agreement and a series of high-level intergovernmental negotiations involving more than 140 countries on a treaty establishing a series of protective measures (including controls on mercury emissions from coal-fired and industrial power plants, as well as the use of mercury in small-scale gold mining). Also, the WHO, in its Global Action Plan, scheduled for 2023–2030 for oral health, stipulates that 90% of countries implement measures to phase out the use of dental amalgam. This is also stipulated in the Minamata Convention [6,8,97]. The Minamata Convention on Mercury entered into force on 16 August 2017, while 114 countries ratified it at the end of 2019. This convention includes a ban on the creation of new mercury mines with the phasing out of existing ones, the phasing out and phasing in of mercury in many products and processes, and the control of greenhouse gas emissions and exposures on land and water [49,54,68,97].

Currently, the proposed World Health Organization interventions cover a wide range, including elimination of mercury mining and its use in gold mining and other industrial processes. They suggest widespread promotion of the use of clean energy sources that do not burn coal. They encourage the transition to mercury-free thermometers and sphygmomanometers in healthcare and the successive implementation of safe use and elimination of mercury-containing products/waste [8,54].

It is worth mentioning that three governments (Gabon, Jamaica, and Sri Lanka) have joined together in a joint $14 million project to eliminate the use of mercury in the formulation in skin-brightening products. Meanwhile, an analysis of more than 300 products from 22 countries by the Zero Mercury Working Group and Biodiversity Institute found that the creams exceeded the allowable limit by up to 100 times (from 93 ppm to more than 33,000 ppm). As a result, the United Nations Environment Program (UNEP), funded by the Global Environment Facility (GEF) and implemented by the World Health Organization and the Biodiversity Research Institute (BRI), was introduced to eliminate these products with its content used to brighten the skin. The project focuses on raising awareness and developing regulations to restrict marketing, production, and trade [6,8,49]. The U.S. Environmental Protection Agency has issued a guide encouraging people to avoid creams that may contain, for example, the term *anti-aging* or *skin-wetting*, as they are a source of exposure to Hg [69].

Interestingly, the Korean National Environmental Health Survey (KoNEHS) monitors exposure to heavy metals, including Hg, in the general population every 3 years. The goal is to continuously assess environmental health at both regional and national levels, evaluating the effects of hazardous substances on human health. And yet, they still remain at a higher level [43] than biomonitoring studies from other regions of the world (e.g., NHANES) [82], the Canadian Health Measures Survey (CHMS) [81], and the German Environmental Survey—GerES) [47].

The UK’s Food Standard Agency (FSA) recommends that pregnant women, women of childbearing age, and children under 16 should avoid eating i.a. and tuna, while children should avoid eating more than two tuna steaks per week. The U.S. Department of Agriculture (USDA) recommends that its residents not consume more than 227 grams of seafood per week. The European Food Safety Authority (EFSA), on the other hand, recommends no more than a 50 g/week consumption of oily fish in children, 125 g in adults, and conservative amounts for pregnant women. Canada recommends up to 150 g for adults, similar to the New Zealand and Australian Standards, except that in children under 6 years, 75 g once every two weeks [6,51]. 

EFSA, meanwhile, together with the Joint Food and Agriculture Organization of the United Nations/WHO Expert Committee on Food Additives (JECFA), has published a scientific opinion on the public health risks of mercury and methylmercury in food. In 2018, it established a tolerable weekly intake for inorganic mercury of 4 g/kg bw, and for methylmercury of 1.3 g/kg bw [23,98]. The European Commission has also issued a series of guidelines for reducing mercury emissions [97,99].

## 9. Prevention

According to the latest knowledge and guidelines, it is important to consider primary prevention, including the broadest possible measures to ensure that our environment and everyday objects are safe for the general population. Self-intervention is also one of the important measures. Educating the general public, as well as health professionals and policy-makers, results in broader efforts to reduce mercury exposure. In fact, very common examples of such educational efforts could be films, games, apps, leaflets, meetings, lectures, or other spots promoting the reduction in everyday products containing toxic metals, including mercury. 

Accordingly, extensive prevention should be carried out, including raising awareness of mercury risk, especially of vulnerable populations, i.e., pregnant women, infants, young parents, or the elderly. A widespread and mandatory series of training courses for healthcare workers should be implemented while promoting proper management and disposal of all mercury derivatives. Keeping constant biomonitoring in people could improve lifestyle habits and environmental factors that affect stress and, consequently, its concentration in the body. Dedicated training of customs agents in identifying products in transit would improve proper control of the sale and distribution of cosmetic products, especially online and in small stores serving populations with darker skin tones. Using the latest laboratory techniques to test for mercury in skin-brightening products would reduce the time it takes to receive approval from the service to produce a cosmetic product. At home, it can be promoted by encouraging consumers to process fish properly, mainly through frying and cooking.

It is absolutely necessary to arouse greater consumer interest, in terms of the safety of selected cosmetic products (mainly complexion brightening), food, and medical devices available on the market, as it is an important factor in the development of the age of diseases.

## 10. Limitations of the Study

More research is needed to confirm the negative effects of all forms of mercury even at the molecular and genetic level, which is of great importance for many disorders. Despite the already well-documented data and information in the literature, it is as if the very fact of its prevalence prompts further research into it, but with the same biomarkers (e.g., only in hair, nails, saliva, blood or urine), identical analyses, and procedures (see Section 7). The designation of the same procedure and the appropriate selection of patients, taking into account the period of exposure and medical history, could be future guidelines for researchers and physicians dealing with the patient. Thus, further analysis in a multifaceted direction seems important to deepen the impact of the knowledge of its toxicity, if only for the sake of the future aging population, as well as newborn children. 

The main aspect that has appeared in practically every available publication has discussed the lack of absolute certainty on whether mercury alone causes disease problems, their mix, and long-term effects. Or whether it is the result of multiple factors, multiple toxic metals, various forms of testing, and definitely a very strong environmental factor. Thus, the effect of Hg cannot be finally and definitively confirmed, as there are tremendous differences in the research methods themselves over even 30 years.

## 11. Conclusions

Exposure to this indestructible element could be fully prevented by using safe and effective mercury-free alternatives. The constant controlling and monitoring of mercury use is a serious public health problem, requiring urgent attention and attentiveness from the governments of all countries and, in the long run, a rapid and concerted response. Thus, it is important to re-analyze the impact of this highly toxic metal on the human body and to prepare as precisely targeted public health interventions as possible. 

The fact of such widespread use as well as its toxicity on the human body alone prompts further and in-depth research in populations with both low and moderate exposure.

There is also an urgent need for integrated public health strategies from possibly all health authorities and additional research focused on reducing the adverse effects of mercury and its derivatives. Because only by combining the forces of scientists, policy-makers, and health professionals can comprehensive interventions with a broad spectrum of reception be created.

## Figures and Tables

**Table 1:** Key characteristics and findings of the reviewed papers.

No.	Author(s)	Country	Topics	Key Point(s)
1	Podgórska et al. (2021) [1]	Poland	Sources in food/packaging	mercury medical products, mercury exposure, mercury health effects
2	Król-Pakulska & Pakulski (2017) [2]	Poland	Mercury in medical products	mercury medical products, sources of mercury food packaging, mercury health effects, mercury toxicity
3	Genchi et al. (2017) [3]	Italy	Health effects, Digestive system	mercury health effects, mercury sources the environment
4	Tian el al. (2023) [4]	China	Health effects, Metabolic and other diseases	mercury health effects, mercury sources the environment, mercury toxicity
5	Ha et al. (2017) [6]	Republic of Korea	Health effects, Digestive system, Guidelines of various international organizations	mercury health effects, mercury sources the environment, mercury toxicity, mercury exposure, mercury global guidelines
6	Adeola (2021) [10]	U.S.	General health effects, Guidelines of various international organizations	mercury health effects, mercury sources the environment, mercury toxicity, mercury exposure, mercury global guidelines
7	Miao et al. (2023) [13]	China	Health effects, Respiratory system, Immune system, Metabolic and other diseases	mercury health effects, mercury exposure, mercury poisoning
8	Ogundipe & Obeng-Gyasi (2025) [14]	U.S.	General health effects	mercury health effects, mercury sources the environment, mercury exposure, mercury poisoning
9	Ogundare & Obeng-Gyasi (2024) [15]	U.S.	Health effects, Neurological system	mercury health effects, mercury sources the environment, mercury exposure, mercury poisoning, sources of mercury food packaging
10	Jorge et al. (2024) [20]	U.S.	Sources in food/packaging, Environment, Health effects, Digestive system, Respiratory system, Reproductive system, Development and its potential disorders, Immune system, Neurological system, Circulatory system, Metabolic and other diseases, Skin, Cancers, Molecular aspect	mercury health effects, mercury vs. in vivo studies, mercury exposure, mercury poisoning
11	Silva-Caicedo et al. (2024) [21]	Spain	Sources in food/packaging	mercury toxicity, mercury exposure, mercury poisoning
12	Dutta & Ruden (2024) [22]	U.S.	Sources in food/packaging, Mercury in medical products, Health effects, Development and its potential disorders	mercury health effects, mercury toxicity, mercury exposure, mercury poisoning
13	Almerud et al. (2021) [23]	Sweden	Sources in food/packaging, Health effects, Metabolic and other diseases	mercury health effects, mercury toxicity, mercury exposure, mercury poisoning, sources of mercury food packaging
14	Ciosek et al. (2023) [24]	Poland	Health effects, Skeletal system	mercury health effects, mercury toxicity, mercury exposure, mercury poisoning
15	Bjørklund et al. (2019) [25]	Norway	Mercury in medical products, Health effects	mercury medical products, mercury sources the environment, mercury toxicity
16	Bjørklund et al. (2020) [26]	Norway	Health effects, Respiratory system, Immune system, Neurological system, Circulatory system, Skin, Molecular aspect	sources of mercury food packaging, mercury medical products, mercury sources the environment, mercury health effects, mercury vs. in vivo studies, mercury toxicity
17	Nyanza et al. (2020) [27]	Canada	Environment, General health effects	mercury health effects, mercury global guidelines, mercury exposure, mercury poisoning
18	Skalny et al. (2022) [28]	Russia	Environment, Health effects, Molecular aspect, Cancers	mercury health effects, mercury global guidelines, mercury exposure, mercury poisoning, mercury vs. in vivo studies, mercury sources the environment
19	Bondy (2021) [29]	U.S.	Health effects, Neurological system	mercury health effects, mercury toxicity, mercury exposure, mercury poisoning
20	Heo et al. (2017) [30]	Republic of Korea	Health effects, Respiratory system, Circulatory system	mercury sources the environment, mercury health effects, mercury toxicity, mercury exposure, mercury poisoning, mercury global guidelines
21	Adetunji & Obeng-Gyasi (2024) [31]	U.S.	Health effects, Metabolic and other diseases	mercury sources the environment, mercury health effects, mercury toxicity, mercury exposure, mercury poisoning
22	Maddheshiya et al. (2024) [32]	India	Health effects, Development and its potential disorders	mercury health effects, mercury toxicity, mercury exposure, mercury poisoning
23	Zag et al. (2017) [33]	Hungary	General health effects	mercury health effects, mercury toxicity, mercury poisoning
24	Kim et al. (2015) [34]	Republic of Korea	Health effects, Respiratory system	mercury health effects, mercury exposure, mercury poisoning
25	Heinrich et al. (2017) [35]	Germany	Health effects, Respiratory system	mercury health effects, mercury exposure, mercury poisoning
26	Verschueren et al. (2020) [36]	The Netherlands	Health effects, Development and its potential disorders	mercury health effects, mercury global guidelines, mercury poisoning
27	Rho et al. (2025) [37]	Republic of Korea	Health effects, Metabolic and other diseases, Guidelines of various international organizations	mercury health effects, mercury sources the environment, mercury exposure, mercury poisoning
28	Kort et al. (2024) [38]	U.S.	Health effects, Reproductive system	mercury health effects, mercury exposure
29	Pamphlett & Kum Jew (2019) [39]	Australia	Health effects, Skeletal System, Molecular aspect	mercury health effects, mercury exposure, mercury toxicity
30	Kim et al. (2022) [40]	Republic of Korea	Health effects, Cancers	mercury health effects, mercury exposure, mercury sources the environment
31	Koh et al. (2019) [41]	South Korea	Health effects, Respiratory system, Skin	mercury medical products, mercury sources the environment, mercury health effects
32	Ijomone et al. (2025) [42]	Nigeria	Health effects, Neurological system	mercury toxicity, mercury exposure, mercury poisoning, mercury health effects
33	Chen et al. (2018) [43]	U.S.	Health effects, Neurological system, Circulatory system	mercury sources the environment, mercury health effects
34	Li et al. (2024) [44]	U.S.	Health effects, Circulatory system	mercury sources the environment, mercury health effects, mercury toxicity, mercury guidelines
35	Jung et al. (2016) [45]	Republic of Korea	Health effects, Circulatory system	mercury sources the environment, mercury health effects, mercury toxicity, mercury exposure
36	Downer et al. (2017) [46]	Spain	Health effects, Circulatory system	mercury health effects, mercury sources the environment, mercury toxicity, mercury exposure, mercury poisoning
37	Cervini-Silva et al. (2021) [47]	Mexico	Health effects, Skeletal system	mercury health effects, mercury toxicity, mercury exposure, mercury poisoning, mercury test methods
38	Rhee et al. (2020) [48]	U.S.	Health effects, Cancer	mercury health effects, mercury toxicity, mercury exposure, mercury poisoning

**Table 2:** Summary of Hg toxicity in individual parts of the human body.

Human Body	Effects of Exposure to Mercury Poisoning
Digestive system [3,6,20,33,49,70]	- Digestive disorders by inhibiting the production of digestive trypsin, chymotrypsin, pepsin. - Ingestion causes severe salivation, abdominal pain, vomiting, indigestion, stomach ulcers, bloody diarrhea, blue–purple borders on the gums, necrosis of the intestinal mucosa, nausea, lethargy, hepatitis, and even death. - Long-term exposure disrupts liver and appendix function.
Respiratory system [13,20,30,34,35,49,50,71,72]	- Exposure causes an inflammatory reaction in the lungs or airways, reducing lung function and making breathing difficult. - Acute vapor poisoning causes pneumonia and bronchitis, pulmonary fibrosis, chest pain, angina leading to death, due to respiratory failure. - Asthma, change in lung function. - In workers, for example, Young’s syndrome.
Reproductive system [20,22,27,34,36,71,72,73,74,75]	- Induce changes in circulating levels of luteinizing hormone (FSH), luteinizing hormone (LH), estrogen, progesterone, inhibin, and estrogens. - Decreased fertility. - In men, it adversely affects spermatogenesis, testicular weight, erectile dysfunction. - In women, ovarian dysfunction, painful and irregular periods (including short, long, irregular cycles), tilted uterus, or premature menopause. - Sexual dysfunctions. - Associated exposure with the incidence of miscarriages, spontaneous abortions, low birth weight, and even stillbirths.
Development and its potential disorders [20,22,32,37]	- Prenatal exposure has been connected to significant impairments in a child’s neurodevelopmental and cognitive abilities (e.g., delayed learning and memory deficits). - After exposure in the fetus cerebral and psychomotor palsy. - A marked decrease in the total brain mass. - Lowering of the child’s intelligence quotient (IQ). - Constant exposure causes higher incidence of spontaneous abortions, stillbirths, premature births, low birth weight, and visible congenital anomalies.
Immune system [13,20,26,49]	- Various types of inflammation. - Constant exposure impairs the immune defense system. - Systemic/local inflammation.
Neurological system [6,15,20,26,29,38,39,40,49,50,65,70]	- Neurological and behavioral disorders. - Mainly manifests as tremor (specific handwriting change), memory loss, insomnia, anxiety, depression, peripheral neuropathy, headaches, impaired concentration, hallucinations, personality changes, cognitive and motor disturbances, and neuromuscular response. - The risk of developing multiple sclerosis (MS), neurobehavioral defects, or anorexia. - Disrupts sensory systems (e.g., retinopathy, visual neuropathy, loss of hearing or vision, reduced sense of smell, abnormal touch). - Negatively on the CNS in children exposed before birth. - Dysfunctions of microglia, astrocytes, and oligodrocytes. - Associated with neurodevelopmental disorders (e.g., autism spectrum disorder (ASD) and neurodegenerative diseases: Alzheimer’s, Parkinson’s). - Causes swelling of astrocytes, resulting in neuronal damage. - Disrupts calcium homeostasis and neurotransmitter metabolism. - *Minamata-byō* diseases.
Circulatory system [3,26,30,40,41,42,76]	- Even low exposure can affect heart rate variability, ischemic heart disease, myocardial infarction, vascular atherosclerosis, myocardial paroxysmal activity, cardiomyopathy, cardiac autonomic function or probable stroke. - May affect endothelial dysfunction as well as thrombosis. - Increases the risk of developing Kawasaki disease (KD). - A potential cause of increased High Risk Category (HRC).
Metabolic and other diseases [4,6,13,23,31,43,49,50,54]	- Exposure leads to kidney disease (increased protein in the urine), increased levels of gamma-glutamyltransferase (biomarker of liver function). - The development of non-alcoholic fatty liver disease (NAFLD), among other conditions. - Disrupts homeostasis, disrupting the absorption, distribution and utilization of essential elements. - Leads to oxidative stress, inflammation and the onset of various liver diseases. - Continuous exposure contributes to renal syndrome, tubular disruption, secondary focal segmental glomerulosclerosis, renal group proteinuria, syncretic nephrotic syndrome, glomerular disease, and membranous nephritis. - Increase in adrocorticotropic hormone, leading to adrenal hyperplas (Addison’s development). - Abnormal functioning of the pancreas with reduced regulation of blood glucose levels.
Skeletal system [24,44,45]	- Influence on bone defects by accelerating at least bone mineral density loss and osteoporosis or osteopathy. - An important role in the pathogenesis of joint and connective tissue disorders, mixed connective tissue disorder, osteoarthritis, rheumatoid arthritis, systemic sclerosis (SSc), systematic lupus erythematosus (SLE), fibromyalgia and Sjogren’s syndrome, among others.
Skin [1,26,49,59,72]	- Poisoning manifests itself as, among other things, redness, rash, facial swelling or even excessive sweating. - On the skin affects the hair, its dryness, porosity, brittleness, thinning and matting, inhibiting the growth phase. - Corrosive to the skin especially (e.g., causing skin rashes, redness, skin discoloration, scarring, reducing skin resistance to bacterial and fungal infections) and to the eyes. - Possible 3 allergic conditions (asthma, allergic inflammation, atopic dermatitis).
Cancers [12,28,46,77]	- Such as kidney, liver, stomach, thyroid, gallbladder, colon, prostate, breast, glioblastoma, lung, basal cell, melanoma, squamous cell and non-melanoma skin cancer. - Mononucleosis factor, participating in leukemia, such as with Hodgkin’s disease.
Molecular aspects [26,28,44,49]	- Molecular mechanisms, e.g., oxidative stress, mitochondrial dysfunction, activation of inflammatory pathways, changes in the sequence of gene regulation by DNA in newborns, and disruption of cell signaling. - Impairs the antioxidant system. - Damages cells such as pancreatic, human embryonic kidney (HEK) and thyroid cell lines. - Is responsible for inflammation or necroptosis and its ability to act as a catalyst for Fenton-type reactions.
